# Dissecting the limited genetic overlap of Parkinson's and Alzheimer's disease

**DOI:** 10.1002/acn3.51606

**Published:** 2022-06-09

**Authors:** Maren Stolp Andersen, Manuela Tan, Inge R. Holtman, John Hardy, International Parkinson's Disease Genomics Consortium, Lasse Pihlstrøm

**Affiliations:** ^1^ Department of Neurology Oslo University Hospital Oslo Norway; ^2^ Institute of Clinical Medicine University of Oslo Oslo Norway; ^3^ Section Molecular Neurobiology, Department of Biomedical Sciences of Cells & Systems University of Groningen, University Medical Center Groningen Groningen The Netherlands; ^4^ Department of Neurodegenerative Disease, Queen Square Institute of Neurology University College London London UK; ^5^ UK Dementia Research Institute at UCL London UK; ^6^ Reta Lila Weston Institute UCL Queen Square Institute of Neurology London UK; ^7^ UCL Movement Disorders Centre University College London London UK; ^8^ Institute for Advanced Study The Hong Kong University of Science and Technology Hong Kong SAR China

## Abstract

Parkinson's disease and Alzheimer's disease show overlapping features both clinically and neuropathologically and elucidating shared mechanisms could have important implications for therapeutic strategies. Evidence for genetic overlap is limited, although enrichment of heritability in genomic regions relevant to microglia has been demonstrated in both disorders. Using summary statistics from genome‐wide association studies, we assessed genetic covariance stratified by cell types and local genetic correlation between Parkinson's and Alzheimer's disease. Significant covariance was observed for neurons only (*p* = 0.00046), and local genetic correlation was significant only in the human leukocyte antigen region (*p* = 1.0e‐05). Our findings support a minor genetic overlap between these two disorders.

## Introduction

Exploring the genetic relationship between complex disorders can shed light on shared pathogenic mechanisms and help generate hypotheses for novel therapies, such as drug repurposing.^1^ A number of methods have been developed to estimate the degree of shared heritability from either individual level genotype data^2^ or summary statistics^3^ from genome‐wide association studies (GWAS). Such tools have been applied to a large number of brain disorders, demonstrating for instance a high degree of shared common risk variants across psychiatric diagnoses.^4^

Parkinson's disease (PD) and Alzheimer's disease (AD) are common neurodegenerative disorders that show overlapping features both clinically and neuropathologically.^5^, ^6^ Perhaps surprisingly, a number of GWAS‐based heritability studies have found little or no evidence of shared genetic architecture between PD and AD.^7^ However, limited general shared heritability on the genome‐wide scale does not rule out the possibility of overlapping genetic factors within specific loci or pathways.

Summary statistics from GWAS may be combined with tissue‐ or cell type‐specific genomic annotations to assess enrichment of common variant heritability.^4^, ^8^ A number of previous studies have implicated microglia in genetic AD risk,^9^, ^10^ and similarly, we recently reported significant enrichment of PD heritability in microglia open chromatin regions.^11^ These findings raise the question of whether elements of the microglia‐specific or neuroinflammatory component of genetic risk may be shared across PD and AD. Furthermore, significant GWAS signals from the human leukocyte antigen (HLA) locus have been reported in both disorders, indicating potentially overlapping immune mechanisms.

To further dissect shared genetic mechanisms across PD and AD, we took advantage of summary statistics from large‐scale GWAS to assess both annotation‐stratified genetic covariance^12^ and local genetic correlation^13^ between the disorders. We also explored how the direction and size of effect for significant GWAS signals reported to date correlate across PD and AD.

## Methods

### GWAS summary statistics

We used the full summary statistics (including 23andMe data) from the 2019 Nalls et al. meta‐analysis of PD GWAS, which included a total of 37,688 cases, 18,618 proxy‐cases, and 1,417,791 controls.^14^ For AD we used summary statistics from the 2019 Jansen et al. meta‐analysis, including 24,087 cases, 47,793 proxy‐cases, and 383,378 controls.^15^ For the overview of significant GWAS signals we used the more recently published data by Wightman et al.^16^ in order to include the maximum number of loci (see Data S1).

### Stratified LD score regression

Linkage disequilibrium score regression (LDSC) is a statistical framework that examines the relationship between GWAS test statistics and LD patterns in a reference sample in order to estimate common variant heritability. In a recently published work, we applied stratified LDSC (s‐LDSC) to partition heritability for PD and assess enrichment across specific cell types.^11^ As a background to the present work, we repeated the same analysis in AD to demonstrate the expected microglial enrichment. We used LD scores estimated from 1000 Genomes data and included HapMap 3 SNPs with an allele frequency above 0.05 (Data Availability). Enrichment was assessed controlling for the effects of 53 functional annotations included in the full baseline model v.1.2.^8^ *p*‐values were calculated based on the coefficient *z*‐score. To define open chromatin in individual brain and immune cell types, we used assay for transposase‐accessible chromatin sequencing (ATAC‐seq) peak data from neurons, oligodendrocytes, astroglia, and microglia^9^ and ATAC‐seq data from monocytes, B‐cells, CD4^+^, and CD8^+^ T‐cells.^17^

### Annotation‐stratified genetic covariance

GNOVA (genetic covariance analyzer) is a framework to estimate annotation‐stratified genetic covariance using GWAS summary statistics and dissect patterns of shared risk that may not be captured by a global test.^12^ We applied GNOVA to PD and AD summary statistics using the same LD score reference and open chromatin annotations as for LDSC above. We used correlation estimates and *p*‐values corrected for sample overlap by GNOVA, Bonferroni‐correcting for eight independent cell types.

### Local genetic correlation

Whereas GNOVA estimates correlation within a functionally defined set of regions spread out across the genome, ρ‐HESS (Heritability Estimator from Summary Statistics) is a method to partition the genome into consecutive LD blocks and assess genetic correlation between two traits locally within each of these blocks.^13^ We applied HESS v0.5.3 to estimate local genetic correlation using the downloadable genome partition files and Europeans' LD reference data from 1000 genomes provided on the HESS webpage (Data Availability) and restricting analysis to HapMap3 SNPs with a minor allele frequency above 0.1. We adjusted for sample overlap as recommended by the ρ‐HESS developers (Data S1). The significance threshold was adjusted for the number of genome partitions tested.

### Visualizing association with PD versus AD for genome‐wide significant SNPs

To compare the magnitude and direction of effect of significant SNPs reported in PD and AD GWAS we generated *z*‐scores from summary statistics, excluding *APOE*. We calculated the Pearson correlation and plotted the results using R.4.0.3.

## Results

**Figure 1:**
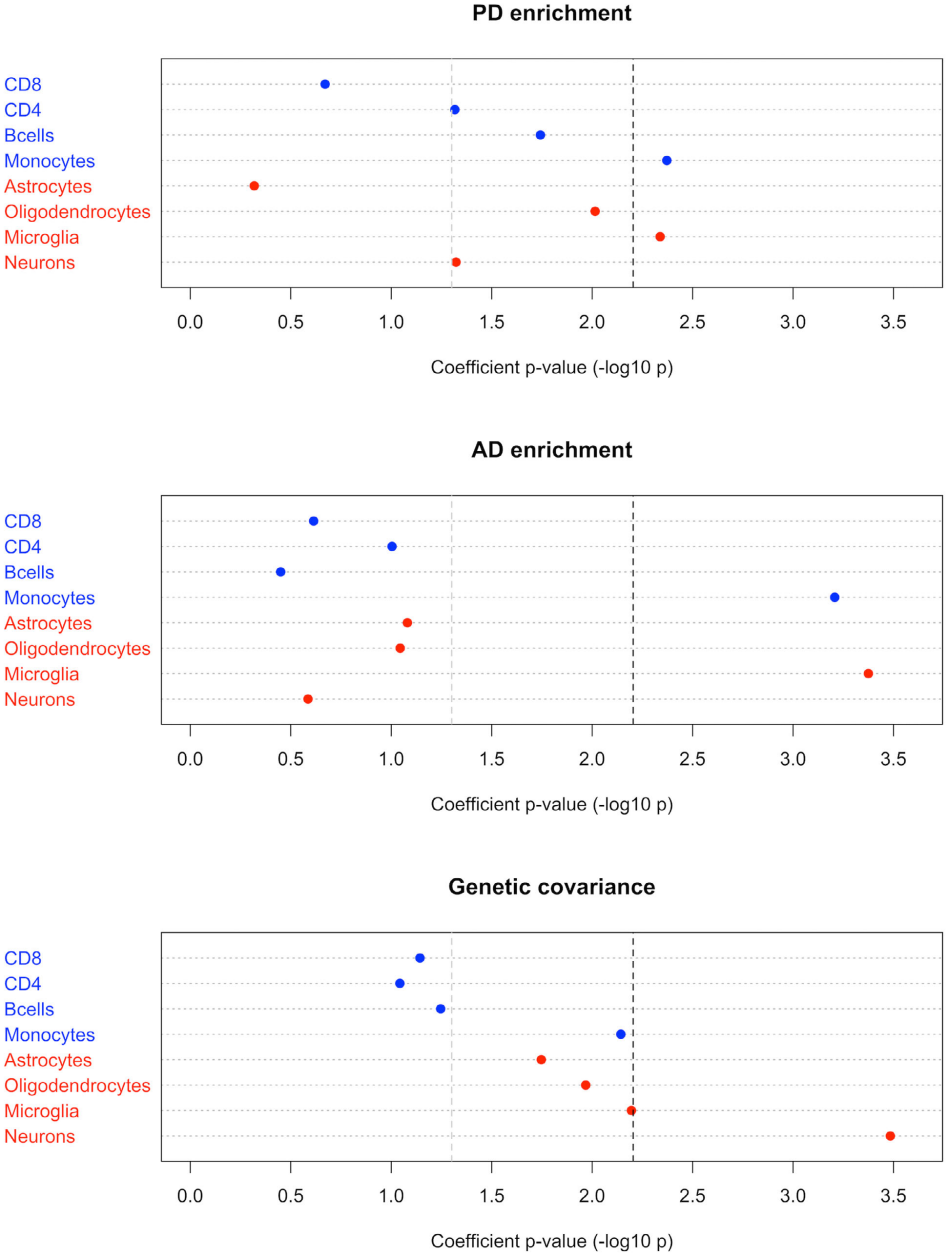
Cell type stratified heritability enrichment and genetic covariance. Results from estimation of heritability enrichment for AD and PD by s‐LDSC and genetic covariance between the two disorders by GNOVA, using open chromatin regions in immune and brain cells as identified by ATAC‐seq to partition the genome. The black dashed line represents a Bonferroni‐corrected significance threshold of *p* < 0.00625, correcting for eight different cell types. AD, Alzheimer's disease; PD, Parkinson's disease; s‐LDSC, stratified linkage disequilibrium score regression; GNOVA, genetic covariance analyzer; ATAC‐seq, assay for transposase‐accessible chromatin sequencing.

Estimated genome‐wide genetic covariance was similar across GNOVA (0.0020, SE = 0.0004, *p*‐value = 4.0e‐5), LDSC (0.0017, SE = 0.0007), and HESS (0.0023, SE = 0.0010). When genetic covariance was stratified by open chromatin in the brain and immune cell types, significant covariance was observed for neurons only (0.00046, SE = 0.0001, *p*‐value = 0.00046) when correcting for multiple testing (Fig. 1).

**Figure 2:**
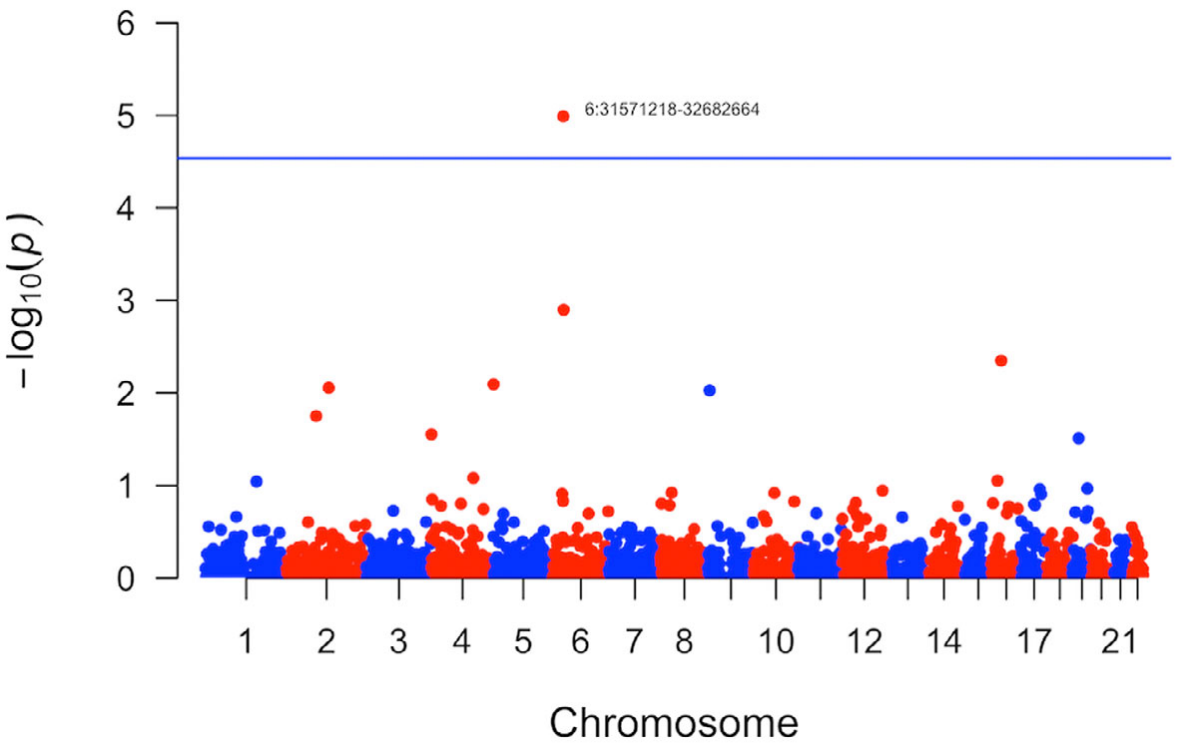
Manhattan plot of local genetic correlation. The figure shows the *p*‐values for genetic correlation between Parkinson's and Alzheimers disease as estimated using ρ‐HESS. The vertical line corresponds to a Bonferroni‐corrected significance threshold of 2.9e‐5, correcting for 1701 independent genomic partitions. ρ‐HESS, heritability estimator from summary statistics.

Local genetic correlation was estimated for 1701 genomic partitions as provided by the tool developer, corresponding to a Bonferroni‐corrected significance threshold of 2.9e‐5. One region passed this threshold, namely chr6:31571218–32682664 (local correlation = 0.00013324, SE = 9.1e‐10, *p*‐value = 1.0e‐05), entailing the *HLA* association signals reported as significant in both PD and AD (Fig. 2).^14^, ^15^ This observation prompted us to also assess local genetic correlation with multiple sclerosis (MS) and attention deficit hyperactivity disorder for comparison (Data S1), where a negative correlation in the HLA region for PD and MS (local correlation = −0.0015, SE = 5.2e‐8, *p*‐value = 6.2e‐11) was the only significant finding (Fig. S1).

**Figure 3:**
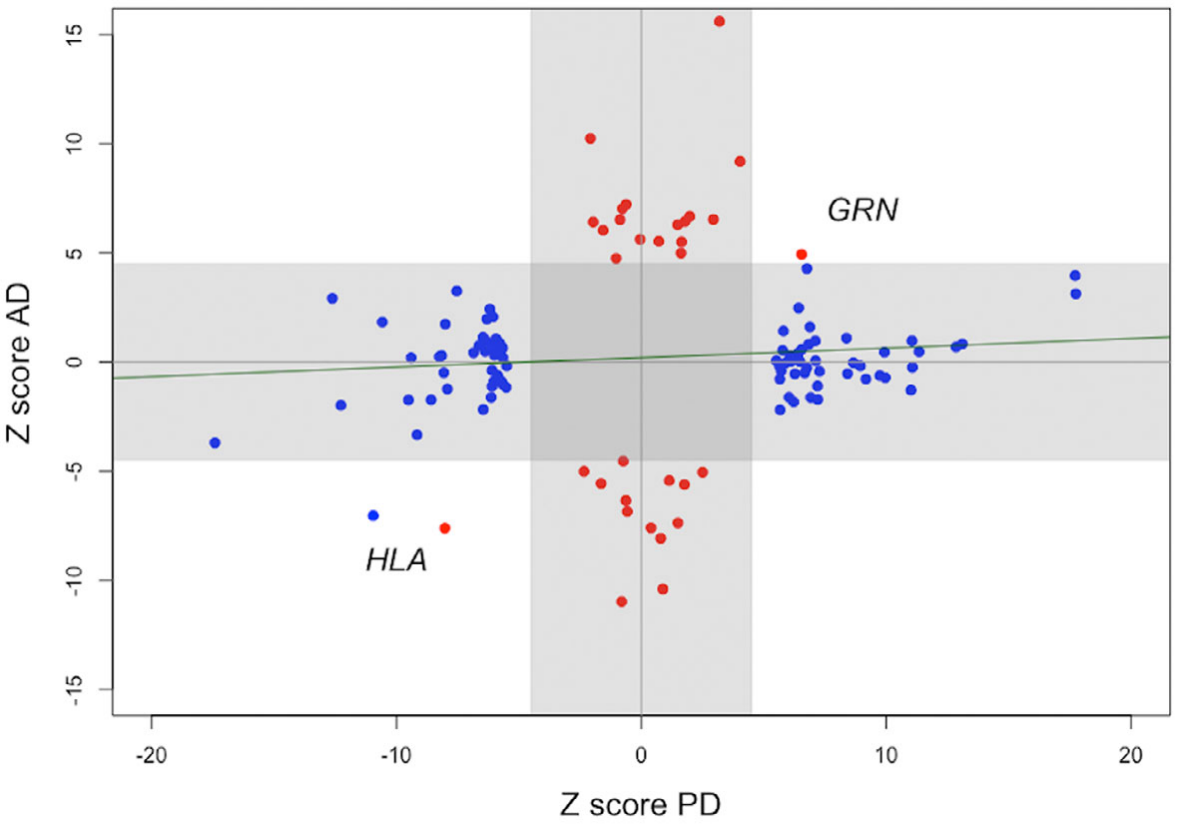
*Z*‐score plot of significant GWAS signals in Parkinson's and Alzheimer's disease. The figure shows *z*‐scores from 90 variants from Nalls et al. (blue) and 38 variants from Wightman et al. (red), excluding the *APOE* locus. Effect sizes in the Wightman data were based on publicly available summary statistics excluding 23andMe data. The gray shaded areas (*z*‐score −4.5 to 4.5) indicate significance in one disorder only, whereas the pleiotropic loci at *HLA* and *GRN* are highlighted in the lower left and upper right white fields. A correlation line shows a nonsignificant relationship between PD and AD *z*‐scores (Pearson correlation 0.08, *p*‐value 0.38). GWAS, genome‐wide association studies; AD, Alzheimer's disease; PD, Parkinson's disease.

Similarly, the *Z*‐score plot (Fig. 3) shows that the GWAS SNPs reported from the HLA locus have the same direction of effect in PD and AD. This is also the case for significant PD and AD SNPs in the *GRN* locus (Table S1), whereas there is no overall correlation of *Z*‐scores (Pearson correlation 0.08, *p*‐value 0.38).

## Discussion

Previous studies have reported limited genetic overlap between PD and AD, but a significant enrichment of heritability in microglia‐related genomic regions has been evident in both disorders. Hypothesizing that PD and AD may show genetic sharing in specific subsets or regions of the genome we analyzed annotation‐stratified genetic covariance and local genetic correlation.

We note as an important caveat that clinical misdiagnosis may drive false positive signals of shared heritability when phenotypes are overlapping,^18^ in particular when “proxy‐cases” with a positive family history are included to further boost sample size.^14^, ^15^ Without neuropathological confirmation, AD GWAS will include some proportion of other dementias. To be interpreted with caution, we detected a significant degree of genome‐wide genetic covariance between AD and PD using GNOVA. Stratifying the analysis by open chromatin regions in brain and immune cell types, we were somewhat surprised to find that despite heritability being enriched in microglia open chromatin in both disorders, the genetic covariance was significant for neurons only. This suggests that although microglial processes are important mediators of genetic risk in both PD and AD, the molecular mechanisms involved are probably largely disease specific.

Analyzing local genetic correlation using ρ‐HESS, the HLA locus was the only region where PD and AD correlated significantly. This result must be interpreted with caution, in particular because correlation on the SNP level does not necessarily imply that the same HLA alleles are involved. Consequently, ρ‐HESS results from this particular region may potentially be non‐specific, as the negative local correlation observed in the HLA region for PD and MS also suggests. GWAS signals from the HLA region have been reported in both PD and AD, and follow‐up studies have performed fine‐mapping, HLA imputation and stepwise conditional regression analyses. A 2017 AD HLA study highlighted the DR15 risk haplotype (DRB1*15:01‐ DQA1*01:02‐ DQB1*06:02), proposing that components of this haplotype could contribute to risk across multiple neurological disorders, including PD.^19^ A recent large PD study, however, found that the HLA association was fully explained by the protective effect of amino acid polymorphisms present in HLA‐DRB1*04 subtypes.^20^ Our genetic correlation results, using data from both disorders, is consistent with the notion that HLA‐related risk is partly shared across PD and AD, although more research is warranted to pinpoint the exact role of different HLA alleles in each diagnosis.

We also highlight concordant effect direction of the *GRN* association signal reported in both PD and AD. *GRN* encodes progranulin and is implicated in frontotemporal lobar degeneration. Further research will be needed to clarify whether these are all genuine signals or if misdiagnosis may contribute to the apparent association of this locus with a broad range of neurodegenerative disorders.

In conclusion, we have detected a significant genetic covariance between AD and PD in neuronal, but not microglial open chromatin regions. We also highlight the common HLA association. Still, the evidence for shared genetic architecture across the two most common neurodegenerative disorders remains limited, underlining that neuroinflammation and neurodegeneration are complex and heterogeneous phenomena following diverse pathways in different disorders.

## Author Contributions

L. P. contributed to conception and design of the study; M. S. A., M. T., I. H., J. H., L. P., and IPDGC contributed to acquisition, analysis and/or interpretation of the data; M. S. A. and L. P. contributed to the drafting of the manuscript and figures; All authors contributed to critical revision of the manuscript.

## Conflict of Interest

Nothing to report.

## Data Availability

23andMe: https://research.23andme.com/dataset‐access/. AD summary statistics: https://ctg.cncr.nl/software/summary_statistics. GWAS Catalog: https://www.ebi.ac.uk/gwas/. Alkes group repository: https://alkesgroup.broadinstitute.org/LDSCORE/. Linkage disequilibrium score regression: https://github.com/bulik/ldsc. GNOVA: https://github.com/xtonyjiang/GNOVA. HESS: https://huwenboshi.github.io/hess/.

## Supporting information


**Data S1.** Supplementary methods.
**Figure S1.** *Z* scores of local genetic correlations.
**Table S1.** SNPs in shared GWAS loci across PD and AD.
