# Supplementary material for: Dissecting the limited genetic overlap of Parkinson's and Alzheimer's disease
Source: Ann Clin Transl Neurol. 2022 Jun 9;9(8):1289–95. doi: 10.1002/acn3.51606 (PMC9380131; doi:10.1002/acn3.51606)
Supplement: Supplementary file 1 — Data S1. Supplementary methods. Figure S1. Z scores of local genetic correlations. Table S1. SNPs in shared GWAS loci across PD and AD. [file ACN3-9-1289-s001.docx]

**Dissecting the limited genetic overlap of Parkinson’s and Alzheimer’s disease**

**Supplementary Material**

**Supplementary Methods**

*Alzheimer’s disease summary statistics*

For AD we used summary statistics from the 2019 Jansen et al. meta-analysis, including 24,087 cases, 47,793 proxy-cases and 383,378 controls.^1^ While our manuscript was in preparation, a larger meta-analysis of AD GWAS was published by Wightman et al.^2^ However, this study reported a lower liability scale single-nucleotide polymorphism (SNP) heritability than the Jansen et al. GWAS (0.031 vs 0.055), potentially due to a more clinically heterogeneous sample. Aiming for the best power to detect genetic overlap with PD, we opted to keep using the Jansen et al. summary statistics for our correlation and covariance analyses. For the overview of significant GWAS signals we used the Wightman data to include the maximum number of loci.

*Adjustment for sample overlap*

As recommended by the ρ-HESS developers, we adjusted for sample overlap using the python-command *estimate_phenocor.py* to estimate phenotypic correlation based on the output from cross-trait LDSC.^3^ The PD and AD meta-GWAS both took advantage of UK biobank proxy-cases and controls.^1, 4^ We assumed a sample overlap corresponding to the smallest number of UK biobank controls reported, namely 328,320 in the Jansen *et al*. article.

*Local genetic correlation analysis of multiple sclerosis and attention deficit hyperactivity disorder*

To further explore the significance of local genetic correlation in the HLA region, we selected an additional two brain disorders, namely multiple sclerosis (MS) and attention deficit hyperactivity disorder (ADHD). Both these disorders have shown limited general shared heritability with PD and AD.^5^ MS has a strong known HLA association, whereas the most recent evidence suggests no role for HLA in ADHD.^6^ We downloaded publicly available summary statistics from the International Multiple Sclerosis Genetics Consortium^7^ (9,772 MS and 17,376 controls) and Demontis et al^8^ (19,099 ADHD and 34,194 controls). We used ρ-HESS as described in the main manuscript to assess local genetic correlation between PD and MS, PD and ADHD, AD and MS and AD vs ADHD.

**Supplementary Figure 1 Z scores of local genetic correlations**

**
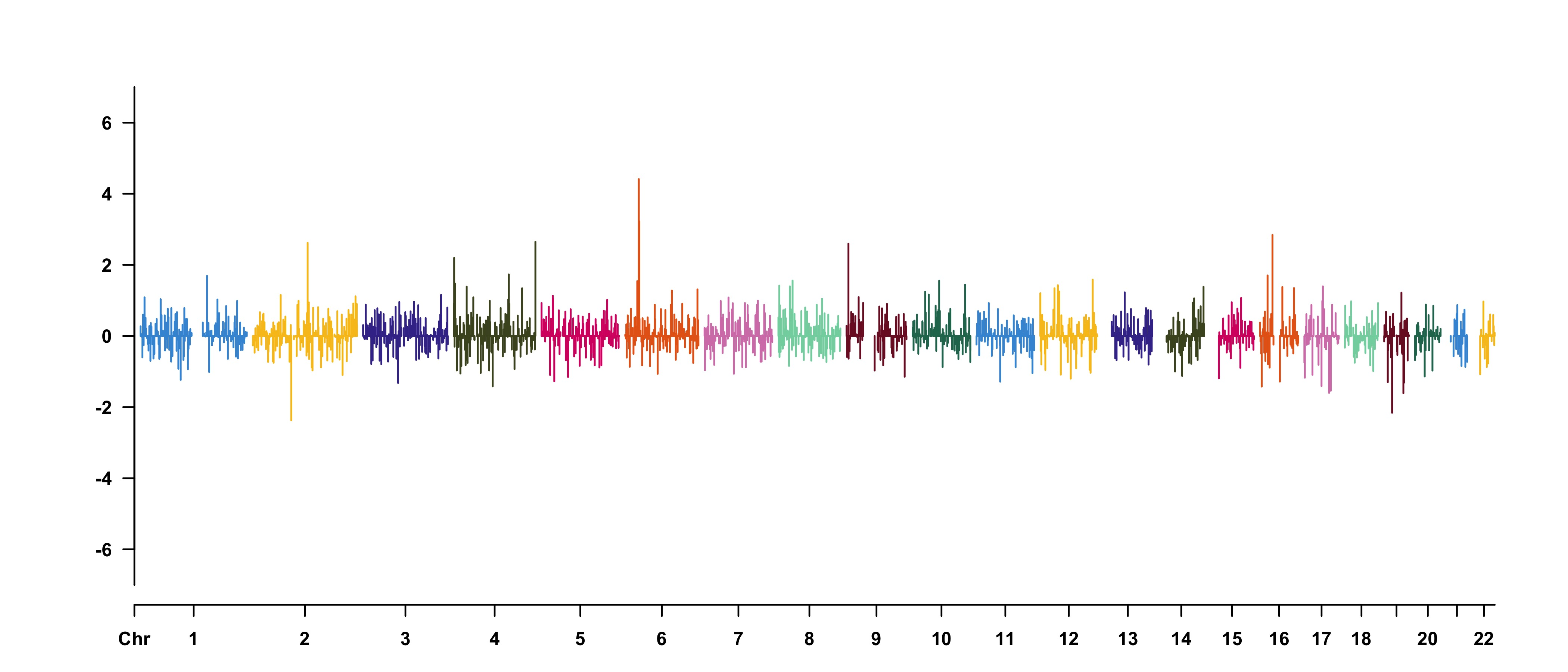
**

PD and AD

**
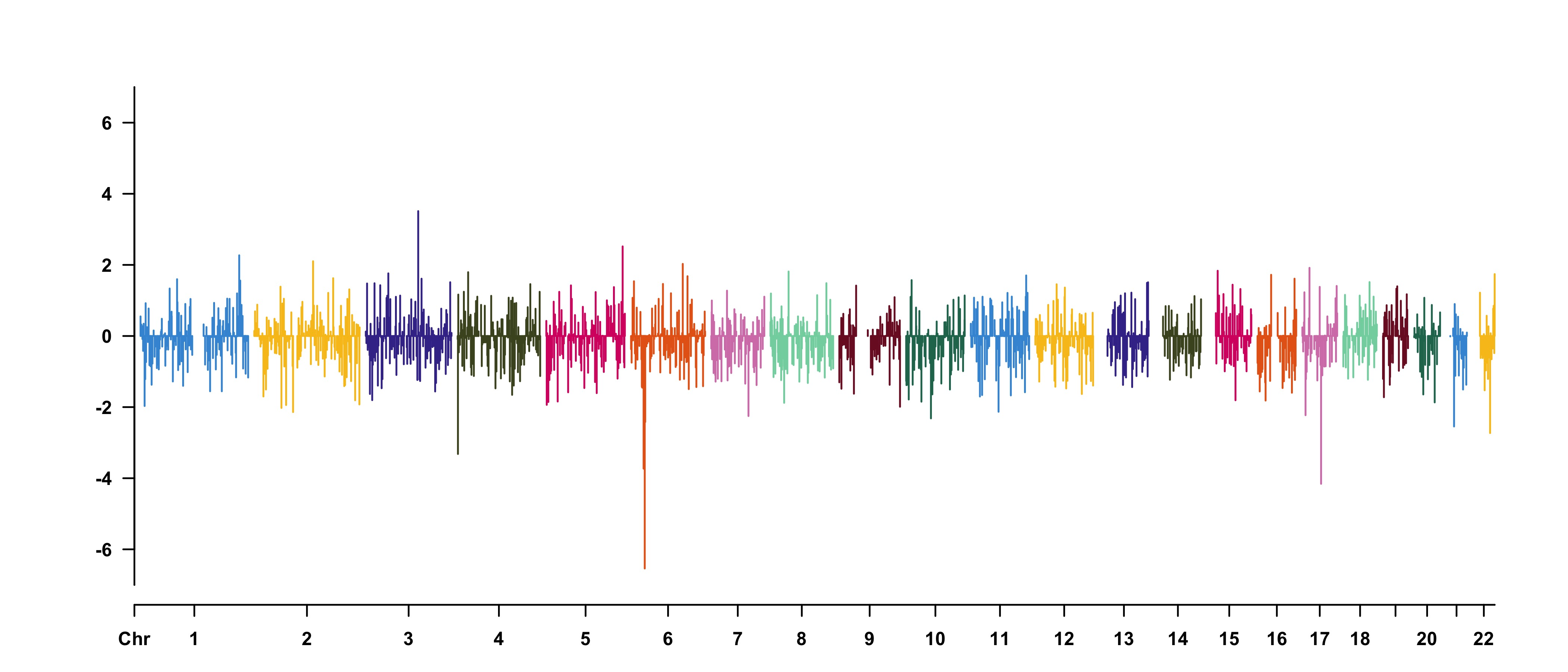
**

PD and MS

.
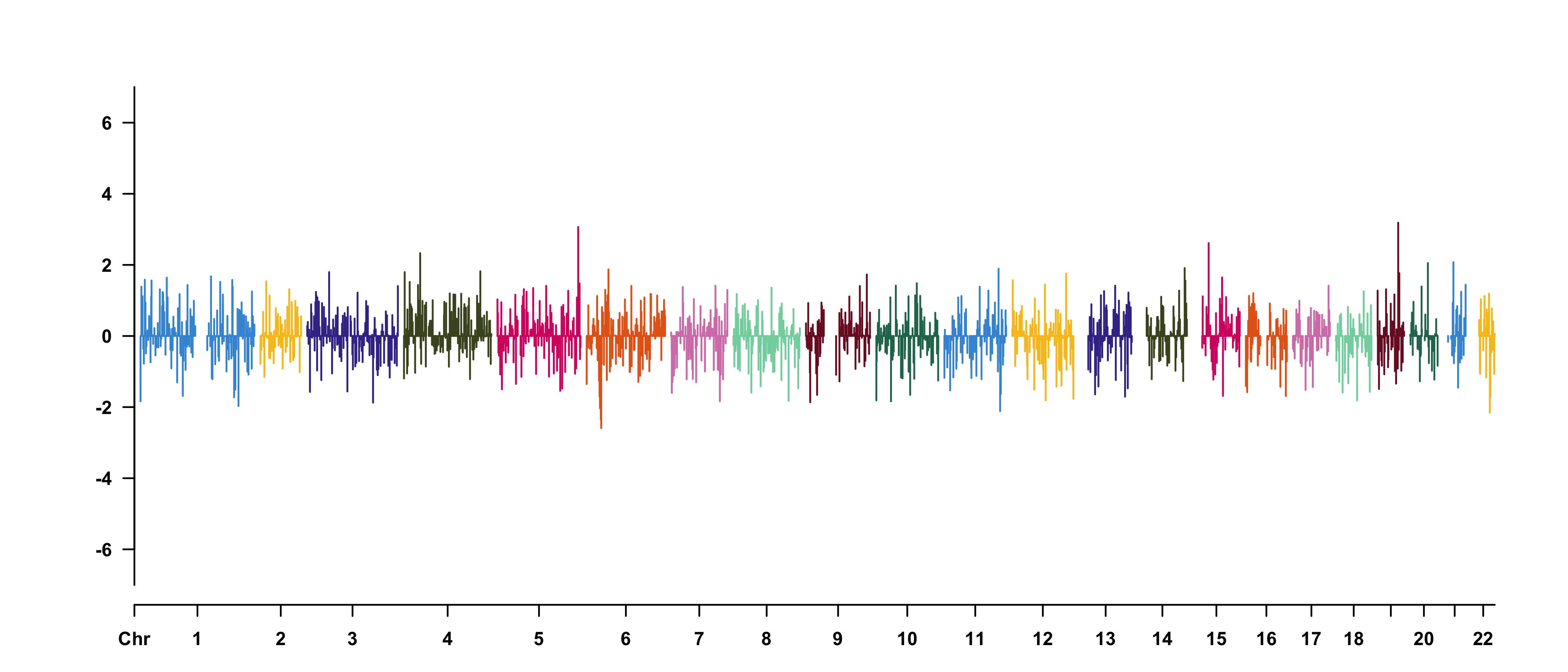


AD and MS


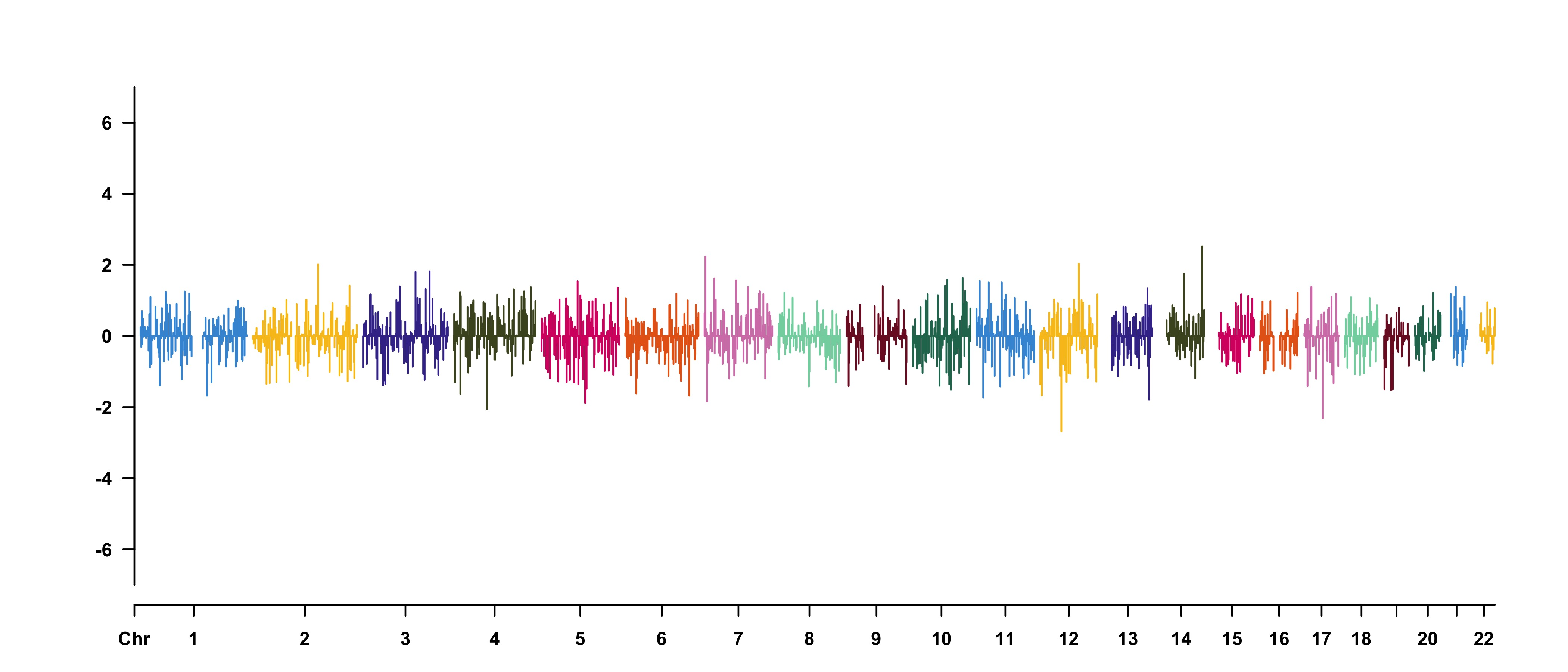


PD and ADHD


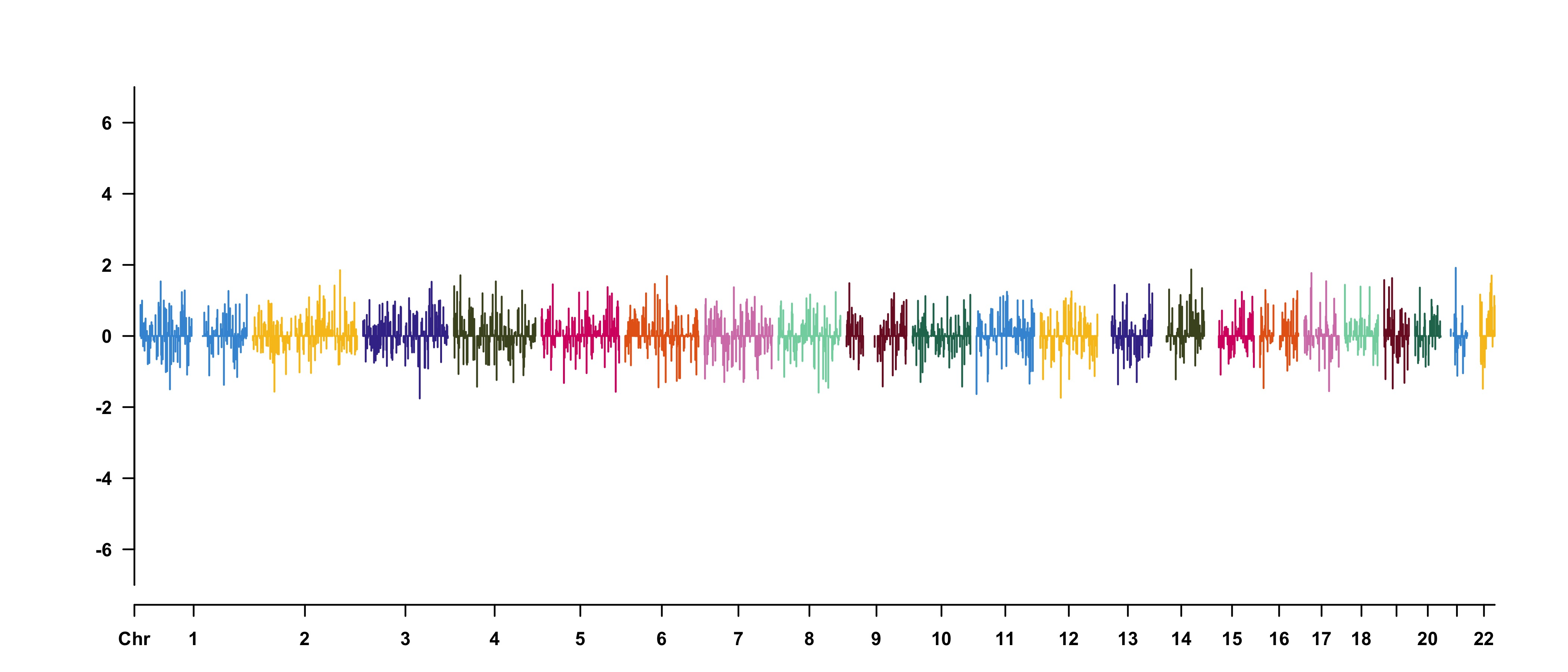


AD and ADHD

The plots show Z scores for local genetic correlation as estimated by ρ-HESS. The only significant signals when adjusting for multiple testing are in the HLA region on chromosome 6; a positive correlation for PD vs AD and a negative correlation for PD vs MS.

**Supplementary table 1 SNPs in shared GWAS loci across PD and AD**

| **SNP** | **Position** | **Effect/other allele** | **Locus** | **Z PD** | **Z AD** | **Reported top-hit** |
| --- | --- | --- | --- | --- | --- | --- |
| rs112485576 | chr6:32578772 | A/C | *HLA* | -11.0 | -7.0 | Nalls et al. PD^4^ |
| rs1846190 | chr6:32583813 | A/G | *HLA* | -8.0 | -7.6 | Wightman et al. AD^2^ |
| rs850738 | chr17:42434630 | G/A | *GRN* | 6.8 | 4.28 | Nalls et al. PD^4^ |
| rs708382 | chr17:42442344 | C/T | *GRN* | 6.5 | 4.93 | Wightman et al. AD^2^ |

The table shows top SNPs in the two genome-wide significant loci that overlap across PD and AD. AD Z scores are from Wightman et al. excluding 23andMe data.

**References**

1. Jansen IE, Savage JE, Watanabe K, et al. Genome-wide meta-analysis identifies new loci and functional pathways influencing Alzheimer's disease risk. Nat Genet 2019;51:404-413

2. Wightman DP, Jansen IE, Savage JE, et al. A genome-wide association study with 1,126,563 individuals identifies new risk loci for Alzheimer's disease. Nat Genet 2021;53:1276-1282

3. Shi H, Mancuso N, Spendlove S, Pasaniuc B. Local Genetic Correlation Gives Insights into the Shared Genetic Architecture of Complex Traits. Am J Hum Genet 2017;101:737-751

4. Nalls MA, Blauwendraat C, Vallerga CL, et al. Identification of novel risk loci, causal insights, and heritable risk for Parkinson's disease: a meta-analysis of genome-wide association studies. Lancet Neurol 2019;18:1091-1102

5. The Brainstorm Consortium, Anttila V, Bulik-Sullivan B, et al. Analysis of shared heritability in common disorders of the brain. Science 2018;360:eaap8757

6. Nudel R, Benros ME, Krebs MD, et al. Immunity and mental illness: findings from a Danish population-based immunogenetic study of seven psychiatric and neurodevelopmental disorders. Eur J Hum Genet 2019;27:1445-1455

7. International Multiple Sclerosis Genetics Consortium, Wellcome Trust Case Control Consortium, Sawcer S, et al. Genetic risk and a primary role for cell-mediated immune mechanisms in multiple sclerosis. Nature 2011;476:214-219

8. Demontis D, Walters RK, Martin J, et al. Discovery of the first genome-wide significant risk loci for attention deficit/hyperactivity disorder. Nat Genet 2019;51:63-75
